# Healthy Lifestyle Practices, Online Health Information–Seeking Behaviors, and Internet Usage Among Pregnant Women: Multigroup Structural Equation Modeling Approach

**DOI:** 10.2196/80637

**Published:** 2026-03-30

**Authors:** Ying Lau, Yueyang Yi, Zebulon To, Sai Ho Wong, Thomas Yuen Tung Lam

**Affiliations:** 1 Nethersole School of Nursing, The Chinese University of Hong Kong, 6-8/F, Esther Lee Building, Shatin, New Territories, Hong Kong, China (Hong Kong), 852 3943 3148; 2 Stanley Ho Big Data Decision Analytics Research Center, The Chinese University of Hong Kong, Hong Kong, China (Hong Kong); 3 Institute of Digestive Disease, The Chinese University of Hong Kong, Hong Kong, China (Hong Kong); 4 Alexandra Hospital, Singapore, Singapore

**Keywords:** healthy lifestyle practices, online health information-seeking behaviors, internet usage, pregnant women, multigroup structural equation model

## Abstract

**Background:**

Singapore is a multicultural society characterized by a diverse array of ethnic groups, including Chinese, Malay, Indians, and others. A considerable percentage of Singaporeans are active users of the internet. The internet has become a significant resource for health education, particularly for women who wish to learn about a healthy lifestyle during pregnancy. However, it is still unclear how pregnant women search for information online, particularly within specific groups.

**Objective:**

This study aimed to explore the relationship between healthy lifestyle practices, online health information-seeking behaviors, and internet usage (IU) among 1905 pregnant women.

**Methods:**

Structural equation modeling (SEM) was used to evaluate the relationships between the appropriate intake of food groups, healthy diet practices (HD), internet for dietary advice (ID), internet for physical activity advice (IP), and IU, based on 5 hypotheses rooted in theoretical concepts. We used a multigroup SEM approach to examine these hypotheses across various ages, ethnicities, BMI, and categories of pregnant groups.

**Results:**

Our results confirmed 5 hypotheses, indicating significant relationships among the variables: appropriate intake of food groups was positively linked to HD (*β*=0.262; *P*<.001); HD was positively linked to ID (*β*=.168; *P*<.001); ID was positively linked to IP (*β*=0.185; *P*<.001); IP was positively linked to IU (*β*=0.190; *P*<.001); and HD was negatively linked to IU (*β*=−0.208; *P*<.001). The multigroup SEM analyses yielded significant differences in Hypotheses 2 and 3 when comparing different age groups (*P*=.009), BMI categories (*P*=.03), and number of pregnancies (*P*=.003).

**Conclusions:**

Our findings offer valuable insights into developing customized online interventions aimed at encouraging a healthy lifestyle during pregnancy.

## Introduction

A healthy lifestyle during pregnancy, including physical activity along with a healthy diet, is beneficial for pregnant women in terms of reducing the risk of maternal and fetal complications [1,2]. With the recent rapid advancement of information and communication technology over the past 2 decades, the internet has emerged as a prominent health education resource for women seeking to learn about dietary and lifestyle modifications during pregnancy [3]. In Singapore, 92.3% of residents are internet users, as indicated by statistics from Global Digital Insights 2025 [4]. Evidence indicates that pregnant women actively use the internet to seek out pregnancy-related information and engage in discussions with peers [5]. Given that the internet serves as a medium for health education, there is a notable lack of investigation into how healthy lifestyles impact online health information-seeking behaviors and internet usage (IU) among pregnant women.

The selection of appropriate food group intake is essential during pregnancy because pregnancy heightens nutritional awareness; thereby, it can help establish healthy dietary practices [6]. Given that the internet serves as a valuable resource for seeking information, pregnant women rely on it to make informed lifestyle choices, encompassing physical activity and healthy eating [7]. A systematic review observed that health information-seeking internet behaviors among pregnant women, especially first-time and young pregnant women, were more likely to find internet-based information [5]. Furthermore, a systematic review revealed that internet use was positively correlated with an increased likelihood of being overweight and obese in the general population [8]. However, it remains unclear whether this conclusion applies to pregnant women.

There are inconsistent findings concerning the relationship between IU and the lifestyle of pregnant women across various countries and ethnic groups, including Iran [9] and Qatar [10]. We found that there is a significant positive relationship between using the internet for healthy behaviors and positive lifestyle changes in Qatari pregnant women [10]. However, 1 study showed no statistical correlation between online health information and a healthy lifestyle in Iranian pregnant women [9]. This discrepancy may be associated with the unique cultural values and beliefs of different ethnic groups. Singapore is a multiracial and multicultural nation, comprising Chinese, Malay, Indian, and various other ethnic groups, all of which contribute to its rich and diverse cultural landscape. Since Singapore is a multicultural country, various ethnic groups may impact these relationships.

This study aimed to investigate the connections among the appropriateness of food group intake, use of online resources for diet and exercise, and IU among pregnant women. A hypothetical model was developed based on protection motivation theory (PMT) [11] and self-control theory (SCT) [12]. The PMT was used to elucidate how pregnant women determine whether to engage in protective behaviors when confronted with a threat by examining the cognitive processes involved in threat and coping appraisal [13]. Pregnant women may pose risks to their unborn babies during pregnancy, yet they are often more inclined to adopt a healthy lifestyle to ensure the safety and development of their child [14]. PMT suggests that pregnant women are more likely than the general population to be motivated by protection, prompting them to actively seek health-related information online regarding a healthy diet and lifestyle [11].

**Figure 1.**
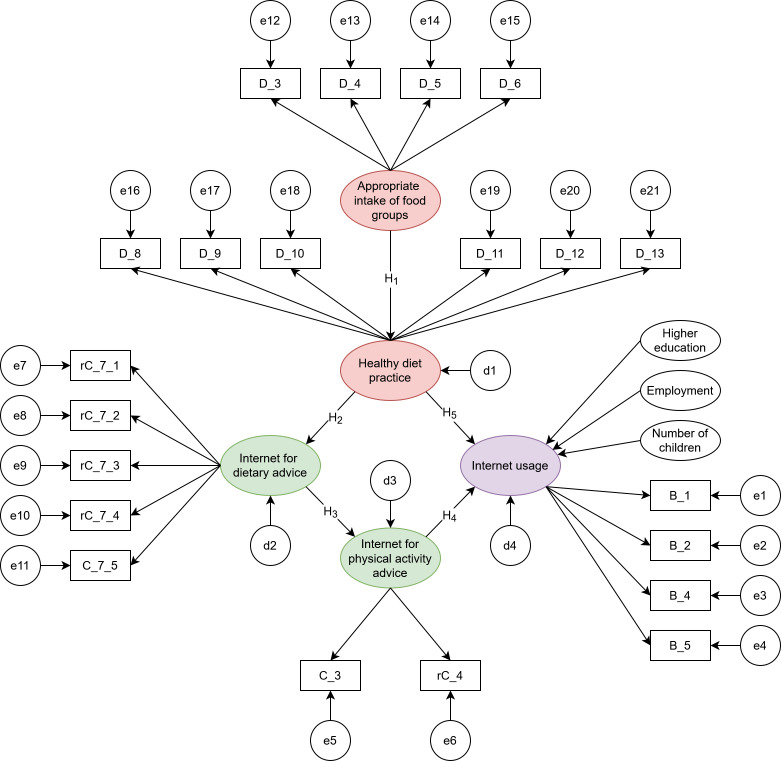
A hypothetical model of the relationships among study variables (appropriate intakes of food groups, healthy diet practice, internet for dietary advice, internet for physical activity advice, and internet usage) in pregnant women. Arrow (→): direct impact; B: Items of Internet Frequency Usage Scale; C: Items of Internet for Physical Activity and Nutrition Scale; D: Items of the Rapid Eating and Activity Assessment for Participant Short Version; d: residue term; e: error term; H_1:_Hypothesis 1; H_2_: Hypothesis 2; H_3_: Hypothesis 3; H_4_: Hypothesis 4; H_5_: Hypothesis 5; r: reversed item. *P < .05; **P < .01; ***P < .001.

According to the SCT, self-control is the ability to regulate current thoughts, feelings, and behavior to secure future benefits [15], which is very important for health and well-being and involves the repeated act of overriding a dominant response [16]. This trait can manifest in behaviors such as adopting an unhealthy lifestyle prior to pregnancy or spending excessive time browsing irrelevant information online [17]. Pregnant women who exhibit high self-control may prioritize an optimal lifestyle by exerting efforts in self-regulation [15,17]. Therefore, pregnant women who intentionally seek the internet for healthy diets may benefit from the deliberative control process, allowing them to more effectively resist the temptation of high-energy foods and irrelevant online information [12]. Based on these theories, we concluded that there could be potential relationships between IU and healthy lifestyles among pregnant women (Figure 1). It is still unclear whether the hypothetical relationships operated in the same way across different groups, particularly among those of varying ages, ethnicities, BMIs, and numbers of pregnant individuals.

Thus, our study sought to assess these relationships based on five hypotheses (H):

1. Hypothesis 1: Appropriate intake of food groups is linked to healthy diet practice
2. Hypothesis 2: Healthy diet practice is linked to seeking dietary advice on the internet
3. Hypothesis 3: The internet’s advice on diet is linked to the pursuit of physical activity advice found on the internet
4. Hypothesis 4: The internet’s advice on physical activity is linked to IU
5. Hypothesis 5: Healthy diet practice is linked to IU

Furthermore, we also compare these hypotheses across various ages, ethnicities, BMIs, and numbers of pregnant groups. Such information is crucial for obtaining insights that can guide future targeted interventions for pregnant women.

## Methods

### Study Design and Data Collection

We conducted this explanatory cross-sectional study among pregnant women who visited 2 antenatal clinics at a university-affiliated hospital in Singapore. There was a public antenatal clinic that served individuals from various economic backgrounds and a private antenatal clinic that catered to those from middle and upper economic classes. We collected data from both clinics, representing a range of socio-economic classes. Data collection occurred while pregnant women were waiting at both clinics. A convenience sampling method was used because of resource constraints. The inclusion criteria were as follows: (1) a minimum age of 21 years, (2) pregnancy in the second trimester (12 to 24 wk of gestation), (3) proficiency in English, and (4) regular access to the internet. A sample size of 1054 was deemed necessary, taking into account a margin of error of 2%, a confidence level of 95%, and a presumed population size of 20,000 [18]. The final anticipated sample size was 1270, which further accounted for a presumed response rate of 83% [19].

Two experienced research assistants (SHW and WWA) recruited all eligible participants in both clinics during the waiting period for their scheduled antenatal follow-up. The research assistants identified moments that were less stressful and more conducive to thoughtful responses, such as right after registration, after checking vital signs, or while participants were waiting for examination results. The research assistants offered a thorough explanation of the study and invited participants to pose any questions they might have. The research assistants made it clear that choosing not to participate would not impact their antenatal care, and participation was entirely voluntary. We allowed sufficient time for each participant to consider their participation in the study. We acquired their written informed consent prior to the commencement of data collection. A self-administered structured questionnaire was designed to be completed in approximately 10 to 15 minutes. This research was carried out in accordance with the STROBE (Strengthening the Reporting of Observational Studies in Epidemiology) guideline to guarantee the quality of the study (Checklist 1).

### Measurements

The study used a package of questionnaires that included demographic and obstetric characteristics, such as age, pre-pregnancy weight and height, race, educational level, employment status, method of conception, number of pregnancies, and number of children, along with 3 validated measures. Classification of BMI groups was based on Singaporean adults [20]: underweight (BMI <18.5 kg/m^2^), normal (BMI 18.5‐22.9 kg/m^2^), overweight (BMI 23‐27.4 kg/m^2^), and obese (BMI ≥27.5 kg/m^2^).

### The Rapid Eating and Activity Assessment for Participants Short Version

The diet was assessed using the 16-item Rapid Eating Assessment for Participant-Shortened version (REAP-S) [21]. The REAP-S questionnaire was designed for adequacy and excess intake of fat, cholesterol, fiber, sugar, and selected food groups [21]. The questionnaire contains 16 items featured with a binary scale (ie, yes or no), a 3-point scale (ie, from usually or often to rarely or never), and a 5-point Likert scale (ie, from not at all to very willing). A higher score indicated healthy diet intake. The scale has been validated with the Block 1998 food frequency questionnaire [21]. Good internal consistency (Cronbach α=.72) [22] and good test-retest reliability (*r*=0.86) were reported [23]. Our study indicated a Cronbach α value of .7.

### Internet for Physical Activity and Nutrition Scale

A 7-item Internet for Physical Activity and Nutrition Scale (IPAS) was adopted to investigate associations between healthy lifestyles and internet use from 1 session of a survey instrument [24,25]. The question types included both dichotomous (yes or no) and ordinal 5-point Likert scales. The items included both positives and negatives. We reversed the negative items before conducting the data analysis. A higher score indicated greater levels of physical activity and adherence to a healthy diet obtained from the internet. We found that this scale demonstrated acceptable internal consistency (Cronbach α=.7).

### Internet Frequency Usage Scale (IFUS)

We created a 6-item Internet Frequency Usage Scale (IFUS) to evaluate the frequency of internet use, drawing on the concepts of the existing IU scale [26]. We adjusted the items to assess the frequency of internet access across various activities, including social media, gaming, emails, and online messaging applications. The sample item was, “On average, how many hours do you spend on social media (Facebook, Twitter, or others) every day?” The scale was designed to be self-completed by answering to what extent they had accessed the internet every day on a 5-point Likert scale from <1 hour (1) to >4 hours (5). The higher the score, the longer the duration of the IU. Cronbach α was calculated to be .7 in this study, indicating an acceptable level of internal consistency.

### Statistical Analysis

R software (version 4.3.2; R Core Team) [27] was used for descriptive and inferential statistical analysis, including Bonferroni-corrected chi-squared tests and 2-sample 2-tailed *t* tests. SAS (version 9.4; SAS Institute Inc) [28] was used for simultaneously examining the hypothesized associations through a structural equation modeling (SEM) approach [29]. Given that there were dichotomous responses in the IPAS, we applied the Satorra-Bentler corrections to the chi-square statistic and standard errors to address the non-normality of our data, in conjunction with the normal-theory maximum likelihood estimator [30]. We addressed missing data using two methods: listwise deletion and imputation. For the demographic data, we opted for listwise deletion to handle the missing information effectively. For each item in the questionnaire, we used imputation to tackle the missing data. Binary response items were imputed using the discriminant function method [31], while all other response items were imputed through regression [32].

A series of exploratory factor analyses was used to identify the structure of variables, while confirmatory factor analyses (CFA) were adopted to test a prespecified theoretical model [33]. Items with a factor loading <0.30 or a communality <0.10 were excluded due to inadequate explanation of the proposed constructs [34]. Modification indices (MI) were used to improve the fitness of models. Goodness-of-fit criteria for the model fit included a ratio of chi-square to its degree of freedom <5, an incremental fit index >0.90, a Tucker-Lewis index >0.90, a comparative fit index >0.90, and a root mean square error of approximation <0.06 [35,36].

A multigroup SEM was performed to compare our 5 hypotheses across different ages (<30, ≥30‐34, or >35), ethnicities (Chinese, Malay, Indian, or others), BMIs (underweight, normal, overweight, or obese), and numbers of pregnant (primigravida or multigravida) groups. We quantified these relationships as standardized coefficients (*β*), estimating them with equal factor loadings across the multigroup by establishing metric equivalence to ensure accurate comparisons through chi-squared tests [37]. A *P* value of .05 indicates statistically significant differences among the multiple groups.

### Ethical Considerations

The National Health Care Group Domain Specific Review Board (NHG DSRB Ref: 2017/00423) conducted a review and granted approval for this study on May 17, 2017. We provided each participant with S $5 (US $3.9) as a token of appreciation for their time. We emphasized the voluntary nature of participants’ involvement and the confidentiality of the information collected. All data were stored in an encrypted file protected by 2-factor authentication. Access to the data was restricted to research team members only. All participants provided written informed consent.

## Results

**Figure 2.**
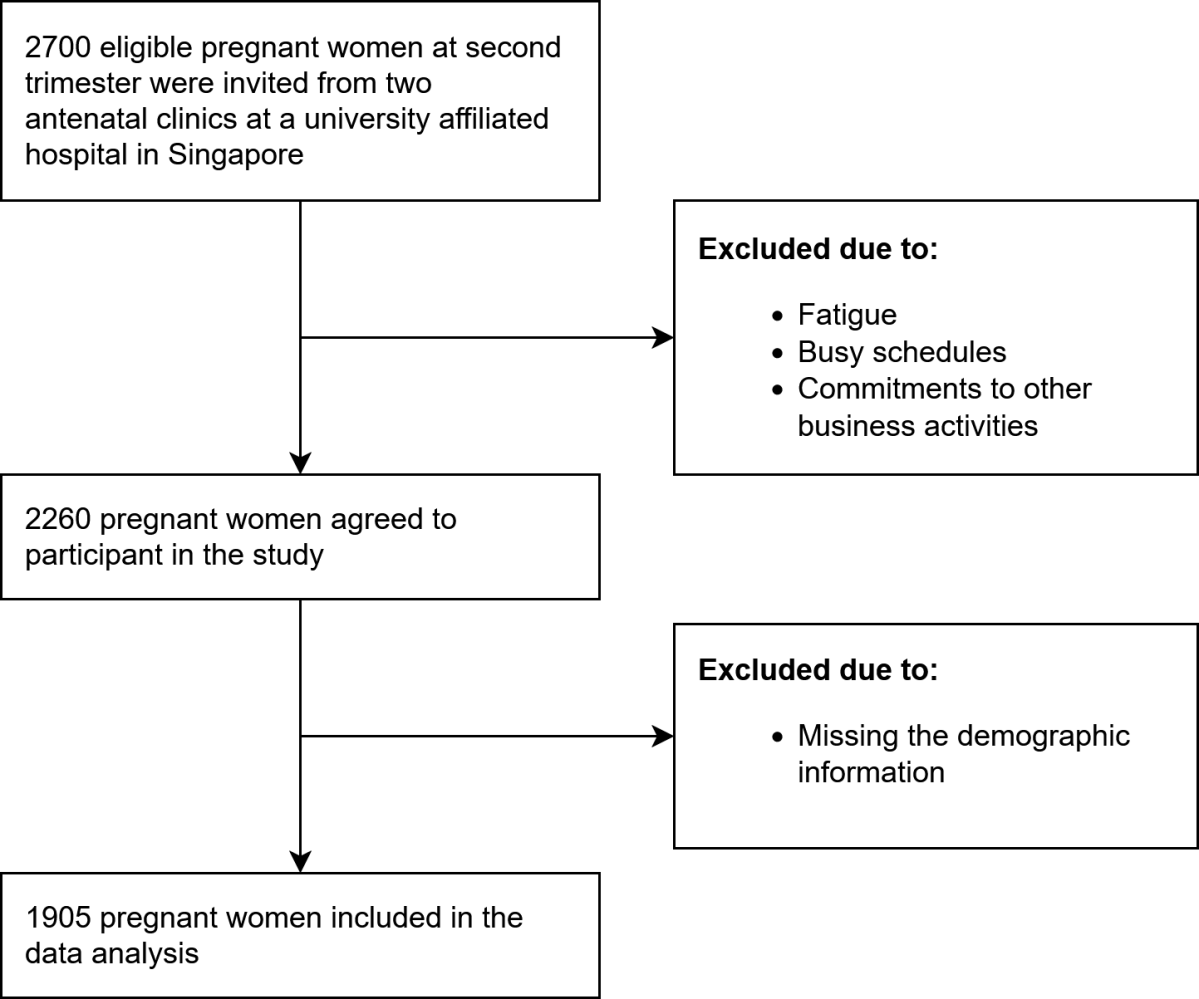
Respondents’ selection process.

We approached 2700 women in total, and 2206 of them agreed to participate in our study (response rate=81.7%). The reasons for nonparticipation included fatigue, busy schedules, and commitments to other business activities. Among these participants, 1905 fully disclosed their demographic information. The process of selection is illustrated in Figure 2. Table 1 summarizes the demographic and obstetrical characteristics of participants. The prevalence of underweight, normal weight, overweight, and obese women was 10.18% (194/1905), 48.56% (925/1905), 28.24% (538/1905), and 13.02% (248/1905), respectively. The mean age was 31.24 (SD 4.05) years. The majority were Chinese (800/1905, 41.99%), followed by Malay (466/1905, 24.46%), Indian (364/1905, 19.11%), and others (275/1905, 14.44%). Main participants obtained bachelor’s degrees or above (1319/1905, 69.24%) and were full-time workers (1483/1905, 77.8%). We observed significant differences in BMI comparison in ethnicity, education, and number of pregnancies and children.

**Table 1.** Demographic and obstetric characteristics of participants by comparisons of underweight, normal, overweight, and obese groups (N=1905).

Demographic variables	Entire (N=1905), n (%)	Underweight^a^ (n=194), n (%)	Normal^a^ (n=925), n (%)	Overweight^a^ (n=538), n (%)	Obese^a^ (n=248), n (%)	*P* value
Age (years), mean (SD)	31.24 (4.05)	30.62 (3.99)	31.30 (4.01)	31.30 (4.11)	31.37 (4.07)	—
Comparisons						
UW^b^ vs NM^c^	—^d^	—	—	—	—	.20^e^
UW vs OW^f^	—	—	—	—	—	.26^e^
UW vs OB^g^	—	—	—	—	—	.03^e^^,^^h^
NW^c^ vs OW	—	—	—	—	—	≥.99^e^
NW vs OB	—	—	—	—	—	≥.99^e^
OW vs OB	—	—	—	—	—	≥.99^e^
Ethnicity groups						
Chinese	800 (41.99)	125 (64.43)	476 (51.46)	164 (30.48)	35 (14.11)	—
Malay	466 (24.46)	34 (17.52)	177 (19.14)	155 (28.81)	100 (40.32)	—
Indian	364 (19.11)	16 (8.25)	138 (14.92)	134 (24.91)	76 (30.65)	—
Others	275 (14.44)	19 (9.79)	134 (14.49)	85 (15.80)	37 (14.92)	—
Comparison						
UW vs NM	—	—	—	—	—	.03^i^^,^^h^
UW vs OW	—	—	—	—	—	<.001^i^^,^^j^
UW vs OB	—	—	—	—	—	<.001^i^^,^^j^
NW vs OW	—	—	—	—	—	<.001^i^^,^^j^
NW vs OB	—	—	—	—	—	<.001^i^^,^^j^
OW vs OB	—	—	—	—	—	<.001^i^^,^^j^
Education levels						
≥ Bachelor’s degree	1319 (69.24)	149 (76.80)	698 (75.46)	348 (64.68)	124 (50.00)	—
< Bachelor’s degree	586 (30.76)	45 (23.20)	227 (24.54)	190 (35.32)	124 (50.00)	—
Comparison						
UW vs NM	—	—	—	—	—	≥.99^i^
UW vs OW	—	—	—	—	—	.02^i^,^h^
UW vs OB	—	—	—	—	—	<.001^i^^,^^j^
NW vs OW	—	—	—	—	—	<.001^i^^,^^j^
NW vs OB	—	—	—	—	—	<.001^i^^,^^j^
OW vs OB	—	—	—	—	—	<.001^i^^,^^j^
Employment status						
Full-time	1483 (77.85)	157 (80.93)	716 (77.41)	423 (78.62)	187 (75.40)	—
Others	422 (22.15)	37 (19.07)	209 (22.59)	115 (21.38)	61 (24.60)	—
Comparison						
UW vs NM	—	—	—	—	—	≥.99^i^
UW vs OW	—	—	—	—	—	≥.99^i^
UW vs OB	—	—	—	—	—	≥.99^i^
NW vs OW	—	—	—	—	—	≥.99^i^
NW vs OB	—	—	—	—	—	≥.99^i^
OW vs OB	—	—	—	—	—	≥.99^i^
Obstetric variables						
Conceptive method						
Spontaneous	1729 (90.76)	184 (94.85)	832 (89.95)	489 (90.89)	224 (90.32)	—
Ovulation drugs	61 (3.20)	2 (1.03)	26 (2.81)	22 (4.09)	11 (4.44)	—
In vitro fertilization	115 (6.04)	8 (4.12)	67 (7.24)	27 (5.02)	13 (5.24)	—
Comparison						
UW vs NM	—	—	—	—	—	.55^i^
UW vs OW	—	—	—	—	—	.62^i^
UW vs OB	—	—	—	—	—	.54^i^
NW vs OW	—	—	—	—	—	.68^i^
NW vs OB	—	—	—	—	—	≥.99^i^
OW vs OB	—	—	—	—	—	≥.99^i^
Number of pregnancies, mean (SD)	1.73 (0.99)	1.56 (0.86)	1.69 (0.99)	1.76 (0.94)	1.98 (1.12)	—
Comparison						
UW vs NM	—	—	—	—	—	.60^e^
UW vs OW	—	—	—	—	—	.10^e^
UW vs OB	—	—	—	—	—	<.001^e^^,^^j^
NW vs OW	—	—	—	—	—	≥.99^e^
NW vs OB	—	—	—	—	—	<.001^e^^,^^j^
OW vs OB	—	—	—	—	—	.03^e^^,^^h^
Number of children, mean (SD)	0.64 (0.88)	0.53 (0.88)	0.59 (0.83)	0.68 (0.86)	0.85 (1.04)	—
Comparison						
UW vs NM	—	—	—	—	—	≥.99^e^
UW vs OW	—	—	—	—	—	.22^e^
UW vs OB	—	—	—	—	—	.001^e^^,^^k^
NW vs OW	—	—	—	—	—	.36^e^
NW vs OB	—	—	—	—	—	<.001^e^^,^^j^
OW vs OB	—	—	—	—	—	<.001^e^^,^^j^

a BMI classification for Singaporean adults.

b UW: underweight.

c NW: normal weight.

d Not available.

e Pairwise 2-sample *t* test with Bonferroni correction

f OW: overweight.

g OB: obese.

h *P*<.05.

i Pairwise *χ2* test with Bonferroni correction

j *P*<.001.

k *P*<.01*.*

The results of exploratory factor analyses and CFAs identified and confirmed a 1-factor structure of IFUS (IU), a 2-factor structure of IPAS (internet for dietary advice and internet for physical activity advice), and a 2-factor structure of REAP-S (appropriate intake of food group and healthy diet practice) after removing inadequate factor loadings. We found that the initial model did not fit well because of model misspecification. Hence, we performed MI by correlating error terms of interpretable covariances based on the evidence. Table 2 presents the median, range, mean, and SD of the IFUS, IPAS, and REAP-S, along with their factor loadings for each item, as determined through the CFAs. The mean (SD) scores for the IFUS, IPAS, and REAP-S were 9.85 (SD 3.62), 11.99 (SD 2.36), and 23.49 (SD 3.17), respectively. The factor loadings for items across 3 scales ranged from 0.327 to 0.942.

**Table 2.** Mean (SD) and median (range) of Internet Frequency Usage Scale, Internet for Physical Activity and Nutrition Scale, and the Rapid Eating Assessment for Participant Shortened Version and subscales, and factor loadings of the items of Internet Frequency Usage Scale, Internet for Physical Activity and Nutrition Scale subscales, and Rapid Eating Assessment for Participant Shortened Version subscales in a series of confirmatory factor analyses.

Scales	Mean (SD)	Median (range)	Structure	Subscales	Mean (SD)	Median (range)	Items	Factor loadings
IFUS^a^	9.85 (3.62)	9 (3.18‐20)	1-factor	IU^b^	9.85 (3.62)	9 (3.18‐20)	B_1	0.646
							B_2	0.777
							B_4	0.71
							B_5	0.327
IPAS^c^	11.99 (2.36)	12 (7-17)	2-factor	IP^d^	4.14 (1.33)	4 (2-7)	C_3	0.525
							rC_4^e^	0.782
				ID^f^	7.85 (1.74)	8 (5-10)	rC_7_1	0.719
							rC_7_2	0.5
							rC_7_3	0.386
							rC_7_4	0.477
							C_7_5	0.942
REAP-S^g^	23.49 (3.17)	24 (10‐31.62)	2-factor	AI^h^	8.67 (1.90)	9 (4‐12.66)	D_3	0.378
							D_4	0.743
							D_5	0.732
							D_6	0.44
				HD^i^	14.83 (2.22)	15 (6‐20.13)	D_8	0.489
							D_9	0.634
							D_10	0.652
							D_11	0.372
							D_12	0.485
							D_13	0.335

a IFUS: Internet Frequency Usage Scale.

b IU: internet usage.

c IPAS: Internet for Physical Activity and Nutrition Scale.

d IP: Internet for physical activity advice.

e r: reversed item.

f ID: Internet for dietary advice.

g REAP-S: Rapid Eating Assessment for Participant Shortened Version.

h AI: Appropriate intakes of food groups.

i HD: Healthy diet practice.

**Figure 3.**
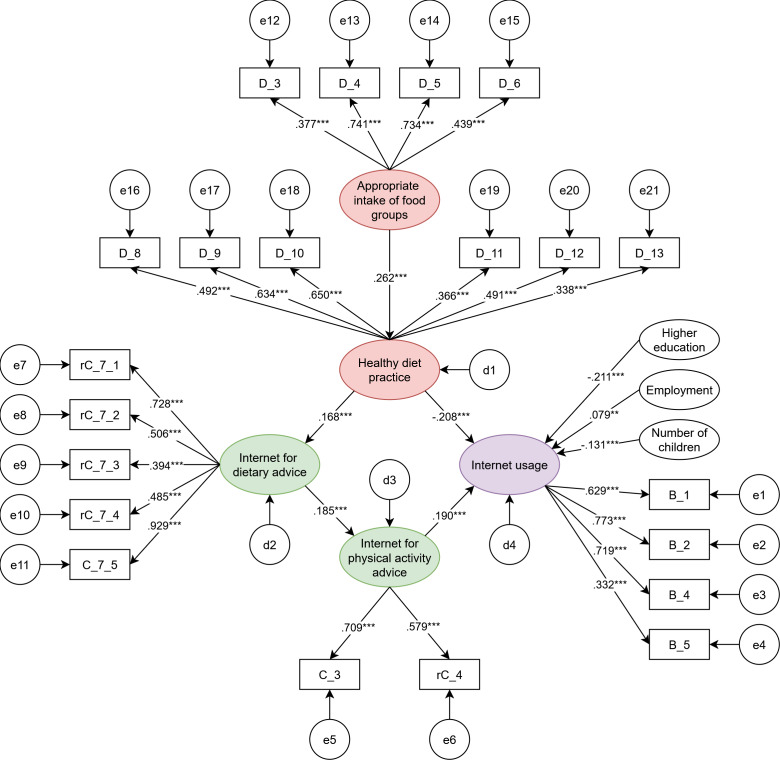
A structural equation model of the relationships among study variables (appropriate intakes of food groups, healthy diet practice, internet for dietary advice, internet for physical activity advice, and internet usage) in pregnant women after adjustment of education level, employment status, and number of children. Arrow (→): direct impact; B: Items of Internet Frequency Usage Scale; C: Items of Internet for Physical Activity and Nutrition Scale; D: Items of the Rapid Eating and Activity Assessment for Participant Short Version; d: residue term; e: error term; r: reversed item. * *P* < .05; ** *P* < .01; *** *P* < .001.

Afterwards, we examined the relationships among appropriate intake of food groups (AI), healthy diet practice (HD), internet for dietary advice (ID), internet for physical activity advice (IP), and IU using an SEM approach, as shown in Figure 3. We also added employment, education levels, and the number of children in the SEM for adjustment. Table 3 presents the goodness-of-fit criteria before and after MI for 3 measures and an SEM. The SEM model demonstrated satisfactory goodness-of-fit indices [35,36], with the following results: *χ*^2^/*df*=3.766 (<5), incremental fit index=0.930 (>0.90), Tucker-Lewis index=0.908 (>0.90), comparative fit index=0.930 (>0.90), and root mean square error of approximation=0.038 (<0.06). Figure 3 suggests that AI was positively linked to HD (*β*=0.262; *P*<.001); HD was positively linked to ID (*β*=0.168; *P*<.001); ID was positively linked to IP (*β*=0.185; *P*<.001); IP was positively linked to IU (*β*=0.190; *P*<.001); and HD was negatively linked to IU (*β*=−0.208; *P*<.001). We concluded that the results supported hypotheses H_1_–H_5_.

**Table 3.** Goodness-of-fit indices of three tools and the final structural equation model.

Models	χ^2^ (*df*)	χ^2^/*df*	IFI^a^	TLI^b^	CFI^c^	RMSEA^d^ (90% CI)	
Internet Frequency Usage Scale							
Initial model	473.724 (10)	47.372	0.775	0.782	0.774	0.121 (0.109-0.133)	
Modified model	12.864 (2)	6.432	0.992	0.992	0.992	0.054 (0.028-0.083)	
Internet for Physical Activity and Nutrition Scale							
Initial model	1109.979 (43)	25.813	0.775	0.753	0.774	0.112 (0.107-0.118)	
Modified model	68.483 (9)	7.609	0.987	0.982	0.987	0.057 (0.044-0.070)	
The Rapid Eating Assessment for Participant Shortened Version							
Initial model	706.915 (103)	6.863	0.821	0.799	0.82	0.054 (0.050-0.058)	
Modified model	169.321 (33)	5.131	0.949	0.939	0.949	0.044 (0.038-0.051)	
A structural equation model							
Initial model	836.018 (222)	3.766	0.93	0.908	0.93	0.038 (0.035-0.040)	

a IFI: incremental fit index.

b TLI: Tucker-Lewis index.

c CFI: comparative fit index.

d RMSEA: root mean square error of approximation.

In Table 4, we performed 4 multigroup SEMs to test these 5 hypotheses by comparing different ages, ethnicities, BMIs, and numbers of pregnant groups. All multigroup SEM analyses yielded significant results concerning the relationship from AI to HD (Hypothesis 1). For the relationship from HD to ID (Hypothesis 2), nonsignificant findings were observed in underweight pregnant women (*P*=.94) aged 30 to 34 (*P*=.05) from other ethnic groups (*P*=.95). There were notable differences between age groups (<30 vs 30–34, *P*=.009) and BMI groups (underweight vs overweight, *P*=.03). For the relationship from ID to IP (Hypothesis 3), no significant results were found in underweight women (*P*=.27) or first-time pregnant women (*P*=.30) from the Indian ethnic group (*P*=.11). We found a significant difference (*P*=.003) between primigravida and multigravida. Although we observed nonsignificant findings in Hypothesis 4 and Hypothesis 5 for specific ethnic and BMI groups, the comparisons between these groups were insignificant.

**Table 4.** Using four multigroup structural equation modeling to compare five hypotheses by age, ethnicity, BMI, and number of pregnancy groups.

Group comparison	H_1_:^a^ AI^b^ →^c^ HD^d^		H_2_:^e^ HD → ID^f^		H_3_:^g^ ID → IP^h^		H_4_:^i^ IP → IU^j^		H_5_:^k^ HD → IU	
	*β* (95% CI)	*P*^l^ value	*β* (95% CI)	*P*^l^ value	*β* (95% CI)	*P*^l^ value	*β* (95% CI)	*P*^l^ value	*β* (95% CI)	*P*^l^ value
All (N=1905)	0.262 (0.201-0.323)	<.001^v^	0.168 (0.110-0.226)	<.001^v^	0.185 (0.127-0.243)	<.001^v^	0.190 (0.124-0.255)	<.001^v^	–0.208 (–0.268 to –0.148)	<.001^v^
Age (years)										
<30 (n=693)	0.235 (0.137-0.334)	<.001^v^	0.282 (0.190-0.374)	<.001^v^	0.135 (0.046-0.225)	.003^u^	0.118 (0.023-0.213)	.02^s^	–0.201 (–0.297 to –0.105)	<.001^v^
30-34 (n=813)	0.251 (0.155-0.347)	<.001^v^	0.087 (0-0.174)	.05	0.178 (0.087-0.269)	<.001^v^	0.257 (0.149-0.364)	<.001^v^	–0.183 (–0.277 to –0.088)	<.001^v^
≥ 35 (n=399)	0.315 (0.179-0.452)	<.001^v^	0.156 (0.021-0.292)	.02^s^	0.262 (0.125-0.400)	<.001^v^	0.192 (0.031-0.352)	.02^s^	–0.201 (–0.342 to –0.060)	.005^u^
Group comparison^m^										
<30 vs 30-34	—^t^	.93	—	.009^u^	—	.75	—	.10	—	.58
30-34 vs ≥ 35	—	.84	—	.28	—	.65	—	.90	—	.54
<30 vs ≥ 35	—	.78	—	.50	—	.47	—	.93	—	.84
Ethnic groups										
Chinese (n=800)	0.277 (0.172-0.383)	<.001^v^	0.178 (0.088-0.268)	<.001^v^	0.137 (0.050-0.224)	.002^u^	0.237 (0.138-0.336)	<.001^v^	–0.207 (–0.307 to –0.107)	<.001^v^
Malay (n=466)	0.224 (0.111-0.336)	<.001^v^	0.225 (0.118-0.333)	<.001^v^	0.245 (0.123-0.366)	<.001^v^	0.068 (–0.059 to 0.195)	.29	–0.055 (–0.170 to 0.059)	.34
Indian (n=346)	0.219 (0.087-0.350)	.001^u^	0.144 (0.003-0.285)	.045^s^	0.110 (–0.026 to 0.246)	0.112	0.118 (–0.035 to 0.271)	.13	–0.248 (–0.398 to –0.099)	.001^v^
Others (n=275)	0.233 (0.058-0.407)	.009^u^	0.006 (–0.166 to 0.179)	.95	0.331 (0.175-0.487)	<.001^v^	0.324 (0.161-0.487)	<.001^v^	–0.216 (–0.374 to –0.057)	.008^u^
Group comparison^m^										
Chinese vs Malay	—	.82	—	.81	—	.21	—	.12	—	.06
Malay vs Indian	—	.45	—	.32	—	.30	—	.72	—	.06
Chinese vs Indian	—	.27	—	.43	—	.91	—	.26	—	.66
Chinese vs Others	—	.46	—	.10	—	.06	—	.21	—	.72
Malay vs Others	—	.63	—	.08	—	.46	—	.03^s^	—	.09
Indian vs Others	—	.86	—	.32	—	.11	—	.06	—	.98
BMI^n^										
UW^o^ (n=194)	0.253 (0.045-0.462)	.02^s^	0.006 (–0.163 to 0.176)	.94	0.106 (–0.083 to 0.295)	.27	0.122 (–0.072 to 0.315)	.22	–0.290 (–0.464 to –0.116)	.001^v^
NW^p^ (n=925)	0.254 (0.163-0.344)	<.001^v^	0.161 (0.077-0.246)	<.001^v^	0.167 (0.080-0.253)	<.001^v^	0.247 (0.155-0.340)	<.001^v^	–0.207 (–0.293 to –0.121)	<.001^v^
OW^q^ (n=538)	0.243 (0.132-0.354)	<.001^v^	0.239 (0.009-0.319)	<.001^v^	0.255 (0.155-0.356)	<.001^v^	0.106 (–0.025 to 0.237)	.11	–0.148 (–0.268 to –0.029)	.02^s^
OB^r^ (n=248)	0.317 (0.159-0.475)	<.001^v^	0.164 (0.130-0.348)	.04^s^	0.217 (0.070-0.365)	.004^u^	0.160 (–0.010 to 0.330)	.06	–0.276 (–0.424 to –0.129)	<.001^v^
Group comparison^m^										
UW vs NW	—	.79	—	.10	—	.63	—	.22	—	.53
NW vs OW	—	.89	—	.41	—	.14	—	.11	—	.42
UW vs OW	—	.86	—	.03	—	.18	—	.96	—	.26
UW vs OB	—	.45	—	.24	—	.34	—	.70	—	.81
NW vs OB	—	.18	—	.64	—	.44	—	.44	—	.65
OW vs OB	—	.23	—	.26	—	.78	—	.62	—	.29
Number of pregnancies										
Primigravida (n=979)	0.273 (0.185-0.360)	<.001^v^	0.200 (0.121-0.279)	<.001^v^	0.043 (–0.038 to 0.123)	.30	0.158 (0.070-0.246)	<.001^v^	–0.204 (–0.287 to –0.122)	<.001^v^
Multigravida (n=926)	0.250 (0.166-0.334)	<.001^v^	0.121 (0.037-0.205)	.005^u^	0.241 (0.162-0.320)	<.001^v^	0.215 (0.121-0.310)	<.001^v^	–0.203 (–0.293 to –0.114)	<.001^v^
Group comparison^m^										
Primigravida vs Multigravida	—	.70	—	.39	—	.003^u^	—	.45	—	.91

a H_1:_ Hypothesis 1.

b AI: appropriate intakes of food groups.

c Arrow (→): direct impact.

d HD: healthy diet practice.

e H_2_: Hypothesis 2.

f ID: Internet for dietary advice.

g H_3_: Hypothesis 3.

h IP: Internet for physical activity advice.

i H_4_: Hypothesis 4.

j IU: Internet usage.

k H_5_: Hypothesis 5.

l *P* value from *t* test.

m *P* value from the *χ2* test.

n BMI classification for Singaporean adults.

o UW: underweight.

p NW: normal weight.

q OW: overweight.

r OB: obese.

s *P*<.05.

t Not available.

u *P*<.01*.*

v *P*<.001.

Table 5 presents a summary of the outcomes related to 5 hypotheses and 4 comparisons across various age groups, ethnicities, BMIs, and numbers of pregnancies. Based on our study, we supported 5 hypotheses within our hypothetical model that was grounded in the PMT [11] and SCT [12]. To extend our knowledge, we discovered that the hypothetical relationships varied significantly across different age and BMI groups for Hypothesis 2, the number of pregnancies for Hypothesis 3, and ethnic groups for Hypothesis 4.

**Table 5.** Summarizing results of hypotheses.

Hypotheses	Entire group	Age group comparisons	Ethnic group comparisons	BMI group comparisons	Primigravida vs multigravida
H1: Appropriate intake of food groups is linked to healthy diet practice.	√	ns^a^	ns	ns	ns
H2: Healthy diet practice is linked to seeking dietary advice on the internet.	√	<30 > 30-34	ns	OW^b^>UW^c^	ns
H3: The internet’s advice on diet is linked to the pursuit of physical activity advice found on the internet.	√	ns	ns	ns	Multigravida > Primigravida
H4: The internet’s advice on physical activity is linked to internet usage.	√	ns	Others > Malay	ns	ns
H5: Healthy diet practice is linked to internet usage.	√	ns	ns	ns	ns

a ns: not significant.

b OW: overweight.

c UW: underweight.

## Discussion

### Principal Findings

This study confirmed the 5 hypotheses among pregnant women. For Hypothesis 1, our findings support the relationship between the intake of food groups and healthy diet practice regardless of different groups of age, ethnicity, BMI, and number of pregnancies. Our results revealed that women who had an appropriate intake of various food groups were more likely to maintain a healthy diet, which aligns with the results of a previous study [6]. The findings aligned with the concepts of the PMT [14], indicating that pregnant women adopted protective behaviors to ensure the safety and development of their unborn babies. The pregnant women perceived poor eating habits as a threat to their pregnancy outcomes and used the internet to assist them in maintaining a healthy diet. They did this by choosing appropriate food groups and engaging in healthy dietary practices [11]. Pregnancy offers a valuable opportunity to embrace health-promoting lifestyle behaviors [38], as this is essential for safeguarding the baby’s health.

For Hypothesis 2, we supported that women who follow healthy dietary practices are inclined to seek dietary advice from the internet. This result is consistent with a previous narrative review [7]. The potential explanation may relate to the fact that most pregnant women find the internet to be the most convenient source for accessing nutritional information tailored specifically for pregnancy, enabling them to connect their healthy eating behaviors [39]. This finding supports the concept of SCT [12], suggesting the pregnant women regulate thoughts, feelings, and behaviors to secure future benefits of their pregnancy by optimizing their lifestyle [15]. Consequently, pregnant women actively sought online health-related information to guide their dietary choices as part of a control process aimed at making informed decisions [12].

However, Hypothesis 2 became insignificant in underweight women aged 30 to 34 when compared to overweight women aged less than 30, and our multigroup comparisons were significant. The finding may suggest that these women are more cautious about their screen time [40]. In contrast, the use of the internet was found to have a significant positive correlation with overweight and obesity [8]. Additionally, younger women were increasingly inclined to use the internet [5].

For Hypothesis 3, our results confirmed that pregnant women who sought online diet advice were more likely to seek online physical advice. One possibility for these observations was that pregnant women sought to ensure a healthy pregnancy and to make informed decisions about their health and the well-being of their unborn children [7]. Physical activity and healthy eating could help pregnant women achieve the recommended gestational weight gain while minimizing the risk of pregnancy complications [41]. This pattern was consistent with the PMT’s theoretical principles [11]; expectant mothers confronted with the threat of pregnancy sought health-related information to ensure a healthy pregnancy [13]. As a result, pregnant women are driven to protect their unborn children by actively seeking internet information about a good diet and lifestyle [14].

Notably, our multigroup SEM comparison revealed a significant difference between primigravida and multigravida. The finding supports previous results indicating that multigravida experience positive outcomes from pregnancy exercises, including improved sleep, reduced stress, decreased back pain, and shorter labor [42]. Another study suggested that primigravida were more likely to be physically inactive than multigravida, as they tended to pay extra caution regarding their pregnancy [43].

For Hypothesis 4, we found that women who received online physical advice were likely to use the internet. The pattern of this result seems to align with findings from a prior study indicating that more than half of pregnant women use the internet to seek information regarding physical activities [10]. Online platforms enable pregnant women to obtain health advice from various sources, prompting them to invest time in seeking reliable information to ensure safe exercise during pregnancy [5], in accordance with the principle of PMT [14].

Although the multigroup comparisons were not significant, we observed that Hypothesis 4 became nonsignificant in both the Indian and Malay groups. Regional variations and cultural differences may account for the observed disparities in physical activity levels during pregnancy, as suggested by a rapid review [44] and 2 studies [45,46]. Malay and Indian Women from the Malay and Indian communities in Singapore each possess distinct sociocultural environments, characterized by significant variations in cultural practices [47]. For both Indian and Malay pregnant women, cultural values place a strong emphasis on rest and modesty during pregnancy, which may restrict physical activities. It was possible that Malay and Indian women might have been dissuaded from participating in antenatal physical activity due to insufficient and contradictory information stemming from cultural beliefs about exercise and physical activity [45,46].

Multigroup comparison also showed that Malay women who sought advice on physical activity from the internet were significantly less likely to be associated with IU compared to women from other ethnic groups. The possibility is associated with socio-economic disadvantages and limited digital literacy [48], particularly affecting certain Malay households in Singapore [47]. In these households, Malay pregnant women faced challenges stemming from restricted digital access. Malay women may exhibit a lack of confidence in interpreting digital information, which aligns with findings observed in Iran [9].

We found that women in the normal-weight group who received online physical advice were significantly more likely to use the internet than those in the underweight, overweight, and obese groups, according to our multigroup analyses. Underweight pregnant women may experience fatigue and decreased motivation for engaging in physical activities [49]. Overweight and obese pregnant women may exhibit lower motivation to participate in online activities, potentially due to feeling overwhelmed by recommendations for lifestyle changes [50].

For Hypothesis 5, our results revealed that participants with healthy diet practice were significantly negatively associated with IU. This may occur because pregnant women who follow a healthy diet are less likely to rely on the internet for dietary information, as they have already established habits and knowledge about nutrition [51]. Additionally, some individuals who maintain a well-balanced diet may prefer to seek guidance from health care professionals, read books, or consult offline resources instead of browsing online [52]. Some pregnant women expressed that the online information available to them failed to instill the confidence required for effectively managing their health [9].

Another potential reason was that they focused on mindful eating and aimed to decrease screen time for the sake of their overall well-being [53]. All usage of the internet is classified as screen time. Screen watching during meals can distract individuals from truly enjoying their food, potentially hindering their ability to recognize satiety signals, which is an essential aspect of intuitive eating [53]. Hence, limiting screens while eating is often associated with mindful and intuitive eating, which can help develop awareness of hunger and satiety signals [54]. Mindful eating may enhance awareness of daily consumption of nutritious and balanced foods, aid in preventing overeating, and promote better digestion [54].

We observed that Hypothesis 5 became nonsignificant in the Malay group. This pattern indicates that cultural factors and personal habits may influence IU [55,56]. A study indicated that individuals in Malaysia express greater concern for their privacy on social networking platforms; consequently, it was probable that they would also have similar privacy concerns when engaging with IU [55]. Personal habits, like limiting screen time or prioritizing offline activities (reading books or participating in outdoor hobbies), could affect IU [56]. Hence, cultural values and personal preferences play a role in influencing IU.

### Strengths and Limitations

This study has several notable strengths. To the best of our knowledge, this study is the first to assess the structural relationships between healthy lifestyle practices, online health information-seeking behaviors, and IU among pregnant women. This evaluation is grounded in the theoretical frameworks of the PMT [11] and SCT [12]. We used multigroup SEM to analyze the structural relationships among various groups. We had a sufficiently large sample size to enhance accuracy, increase statistical power, and bolster confidence in the results.

However, we recognized several limitations in this study. First, because the study is cross-sectional, the findings indicate a temporal relationship rather than a cause-and-effect relationship. Second, using a self-administered questionnaire may introduce recall, informational, and social desirability biases. Furthermore, the reliance on self-reported dietary data lacked objective validation, which could address issues such as portion size misestimation and variability in dietary habits from day to day. Third, since this study only included pregnant women who had made 2 antenatal clinic visits at a hospital, it may not be generalizable to the whole population of pregnant women. Fourth, our study used convenience sampling, which may restrict representativeness and introduce selection bias due to the potential for sampling error. Fifth, while we used validated tools for measuring study variables from other countries, it was essential to culturally adapt them for the local population. Finally, the participants were restricted to women who were proficient in English, which may have resulted in the exclusion of non-English-speaking women. This limitation could potentially affect the generalizability of the findings.

### Implications

Our findings have several important implications. A positive relationship exists between healthy lifestyle practices and online health information-seeking behavior, highlighting the specific needs of pregnant women to seek online information that supports their healthy lifestyle choices. Our findings provide important insights into the effectiveness of online interventions designed to promote a healthy lifestyle during pregnancy. A negative relationship exists between healthy dietary practices and IU. This observation highlights the importance of credible online information, as well as cultural values and personal preferences, in shaping how pregnant women use the internet. Monitoring online content by regulatory bodies is important [57]. Health care professionals ought to be equipped to assist pregnant women with retrieving, interpreting, and using online resources [9]. Various age groups, BMI categories, ethnicities, and numbers of pregnancies exhibited alterations in these relationships. These findings assist in designing targeted interventions tailored to specific groups.

### Future Research

Future research should adopt a longitudinal multisector design to examine the causal relationships between study variables and enhance the generalizability of the findings. Future research could explore the use of objective measurement tools and probabilistic sampling techniques to reduce biases and errors. To gain a comprehensive understanding of healthy lifestyle practices, online health information-seeking behavior, and IU among pregnant women, there is a need for additional qualitative studies. Future research should explore healthy eating and IU among pregnant women during the first and third trimesters, thereby broadening the understanding beyond the second trimester.

### Conclusions

Our study confirms 5 hypotheses and reveals that there are significant structural relationships between healthy lifestyle practices, online health information-seeking behaviors, and IU among pregnant women. The multigroup SEM indicated that age, BMI, ethnicity, and number of pregnancies play a role in affecting these hypotheses. Future research should explore the potential for a longitudinal study to examine causal relationships between the appropriate intake of food groups, adherence to healthy dietary practices, online dietary advice, online physical activity, and IU.

## Supplementary material

10.2196/80637 Checklist 1 STROBE checklist.

## Abbreviations

AI: appropriate intake of food groups
CFA: confirmatory factor analyses
HD: healthy diet practice
ID: internet for dietary advice
IFUS: Internet Frequency Usage Scale
IP: internet for physical activity advice
IPAS: Internet for Physical Activity and Nutrition Scale
IU: internet usage
MI: modification indices
PMT: protection motivation theory
REAP-S: Rapid Eating Assessment for Participant-Shortened version
SCT: self-control theory
SEM: structural equation modeling
STROBE: Strengthening the Reporting of Observational Studies in Epidemiology
